# LncRNA OGFRP1 acts as an oncogene in NSCLC via miR-4640-5p/eIF5A axis

**DOI:** 10.1186/s12935-021-02115-3

**Published:** 2021-08-13

**Authors:** Xiaojing Liu, Na Niu, Pibao Li, Liping Zhai, Ke Xiao, Wendan Chen, Xuewei Zhuang

**Affiliations:** 1 Department of Clinical Laboratory Medicine, Shandong University Qilu Hospital, Jinan, 250012 China; 2 Department of Pediatrics, Shandong Provincial Hospital Affiliated to Shandong First Medical University, Jinan, 250021 Shandong China; 3 Intensive Care Unit, The Third Hospital of Shandong Province Affiliated To Shandong University, Jinan, 250041 China; 4 Shandong Province Endemic Disease Control Institute, Jinan, 250014 China; 5 Department of Clinical Laboratory Medicine, The Third Hospital of Shandong Province Affiliated To Shandong University, #12 Wuying Shan Zhong Road, Tianqiao District, Jinan, 250041 China

**Keywords:** lncRNA OGFRP1, NSCLC, Proliferation, eIF5A

## Abstract

**Background:**

Long noncoding RNAs (lncRNAs) OGFRP1 is up-regulated in endometrial cancer and cervical carcinoma, and OGFRP1 suppression inhibits the malignant behavior of cancer cells. Here, we evaluated the expression pattern, biological function and potential mechanism of OGFRP1 in non-small cell lung cancer (NSCLC).

**Methods:**

The expression of target genes in 25 pairs of clinically collected NSCLC and normal lung tissue samples was detected by qRT-PCR or western blot. We screened the siRNA (siOGFRP1) to down-regulate the expression of OGFRP1 in A549 and H1299 cells. The biological function of A549 and H1299 cells were examined by CCK8, wound healing and transwell assays. The molecular mechanism of OGFRP1 was further explored.

**Results:**

The expression of OGFRP1 in NSCLC tissues were higher than that in normal lung tissue. siOGFRP1 inhibited the proliferation, migration and invasion of A549 and H1299 cells. In addition, the expression of EMT-related and apoptosis-related proteins was changed by siOGFRP1 transfection. OGFRP1 can directly interact with miR-4640-5p, and siOGFRP1 increased the level of miR-4640-5p. Moreover, miR-4640-5p could directly bind to the 3’ UTR region of eIF5A mRNA. eIF5A was highly expressed in NSCLC tissues, and predicted a poor prognosis. In addition, the expression of miR-4640-5p and eIF5A in NSCLC tissues were negatively correlated, while the expression of OGFRP1 and eIF5A were positively correlated. Knockdown of OGFRP1 inhibited the expression of eIF5A, while transfection of miR-4640-5p inhibitor up-regulated the expression of eIF5A.

**Conclusions:**

Taken together, we demonstrated that down-regulation of OGFRP1 inhibited the progression of NSCLC through miR-4640-5p/eIF5A axis.

## Introduction

Lung cancer is a serious life-threatening disease, causing 27% of cancer-related deaths [1]. Non-small cell lung cancer (NSCLC) accounts for 85% of all lung cancer cases [1, 2]. Despite the increasingly desperate efforts on therapeutic technologies to against NSCLC in the past few decades, the survival rate of NSCLC is still low, with ~ 13% (all stages combined) within 5 years [3]. In recent years, with the development of sequencing technology, the cost and speed of sequencing have been greatly improved. Combined with the development of tumor biology, personalized therapy, which is characterized by gene diagnosis and molecular targeted therapy, has become a promising treatment for NSCLC [4–6]. It is increasingly important to find new molecule targets and identify the related action mechanisms for early diagnosis and treatment of NSCLC.

Long noncoding RNAs (lncRNAs) are a category of RNAs longer than 200 bp without protein coding activity [7, 8]. Currently, thousands of lncRNAs have been identified by the ENCODE project and GENCODE annotation. However, the corresponding functional annotations of lncRNAs are extremely insufficient, partly due to their low expression, high tissue specificity and narrow time frames [9–15]. However, current studies suggest that lncRNAs are involved in nearly all biological processes, including cancer cell proliferation, apoptosis, migration and invasion through chromatin remodeling and histone modification, epigenetic modification or sponge effect [16–19]. According to reports, several lncRNAs are important regulators in the progression of NSCLC. For example, LncRNA-PAGBC promoted cell proliferation and metastasis of human gallbladder cancer (GBC) in vitro and in vivo by sponging tumor suppressive microRNAs miR-133b and miR-511 [16]. LncRNA ANRIL functions as an oncogene by interacting with c-Myc in NSCLC [20]. LncRNA FEZF1-AS1promoted tumor progression by inhibiting E-cadherin and modifying WNT pathway in NSCLC [21].

Homo sapiens opioid growth factor receptor pseudogene 1 (OGFRP1), with 1201 nucleotides in length, is a recently identified lncRNA located on chromosome 22q13.2. OGFRP1 is found to be up-regulated in endometrial cancer [22] and cervical carcinoma [23]. Furthermore, OGFRP1 suppression inhibits the malignant behavior of the endometrial cancer cells (Ishikawa) [22], hepatocellular carcinoma cells (Hep3B) [24], cervical carcinoma cells (C33A and SiHa) [23], gestational choriocarcinoma cells (JEG3) [25] and human coronary artery endothelial cells (HCAECs) [26]. However, the expression pattern, biological function and potential mechanism of OGFRP1 in NSCLC have not been investigated. Although, Ding and Liu analyze the RNA-seq data of 551 lung adenocarcinoma (LUAD) patients downloaded from The Cancer Genome Atlas (TCGA), and find that OGFRP1 as an interesting factor involves in the LUAD [27]. Tang et al. find that OGFRP1 regulates LYPD3 expression by sponging miR-124-3p and promotes NSCLC progression [28].

In this study, we used siOGFRP1 to investigate the role of OGFRP1 in NSCLC. Then we examined the changes of miR-4640-5p/eIF5A axis to explain the action mechanism of OGFRP1.

## Materials and methods

### Tissue collection

Tissue specimens were collected from 25 patients diagnosed with LUAD who underwent surgical resection at Shandong University Qilu Hospital. None of the patients suffered from malignant tumors or serious diseases other than LUAD, and had not received radiotherapy or any other treatment before surgery. Participants provided written informed consent before the start of the experiment.

### Cell culture and transfection

Human normal bronchial epithelial cell line 16-HBE and NSCLC cell lines (A549, SPC-A-1, SK-MES-1, NCI-H520, 95D and H1299) were purchased from the Type Culture Collection of the Chinese Academy of Sciences (Shanghai, China) and cultured in RPMI-1640 medium (Gibco, USA) supplemented with 10% FBS (Hyclone, USA), 100 U/ml penicillin and 0.1 mg/ml streptomycin at 37 °C with 5% CO_2_ atmosphere.

Lipofectamine2000 liposome was used to transfect siRNA or plasmid into cells following the instructions. siRNAs targeting to OGFRP1 (siOGFRP1) were designed and synthetized (RiboBio, Guangzhou, China). The sequences of siRNAs were as follows:

siRNA1: 5′-GGTGTTCACATGGCAGTAA-3′; siRNA2: 5′-GGATACTGAGAGTGCACAA-3′; siRNA3: 5′-GCATTGACATGTTTGGCAT-3; NC: 5′-UUCUCCGAACGUGUCACGUTT-3′. miR-4640-5p mimic (UGGGCCAGGGAGCAGCUGGUGGG) and inhibitor (ACCCGGUCCCUCGUCGACCACCC) were purchased from RiboBio (miR10019699-1–5, Guangzhou, China). The cDNA of eIF5A was synthesized by GENEWIZ and cloned into the pcDNA3.1 expression vector (GenePharma, Shanghai, China).

### qRT-PCR

Total RNA was extracted by using TRIzol (Invitrogen) according to the manufacturers’ instructions. The cDNA was formed by using EasyScriptTM Reverse Transcriptase (TransGen Biotech Co., Ltd., Beijing, China). The expression of target was further detected by using the SYBR Premix Ex Taq II (Takara Bio, Dalian, China) and an FTC-300 Real-Time Quantitative Thermal Cycler (Funglyn Biotech Inc., Shanghai, China). The relative quantification was identified by the 2^−∆∆Ct^ method. GAPDH and U6 were used as an internal reference.

Primers used are as follows: OGFRP1 sense: 5’-TGGCTGCCCACAAGATAATG-3’, anti-sense: 5’-GCCTCCCATCAAAAGCTCCT-3’; GAPDH sense: 5’-TCAATGTCGGCGCCTATTTC-3’, miR-4640-5p antisense: 5’-CACCCTGTTGCTGTAGCCAAA-3’; miR-4640-5p sense: 5’-TGGGCCAGGGAGCAGCTGGTGG3’; U6 sense: 5’-CTCGCTTCGGCAGCACA.

### CCK8 assay

After 24 h of transfection, the cells were digested, resuspended and counted. 1000 cells were planted in each well of a 96-well plate. Cell viability was measured every 24 h. For testing, the old medium was removed, and 100 μl of fresh medium containing 10 μl of CCK8 reagent (Beyotime Biotechnology, Shanghai, China) was added to each well of the 96-well plate, and incubated at 37 °C for 2 h. Then the OD value at 450 nm was measured to draw the proliferation curve.

### Wound healing migration assay

After transfection for 24 h, cells were cultured to the appropriate confluence and scratched by a sterile pipettes tip. After washed by PBS, cells were cultured for 24 h in serum-free medium and photographed at 0 h and 24 h. An average of five random widths fields of each wound was measured for quantification.

### Transwell assay

The 8 μm pore transwell chamber was coated with Matrigel. The cells that had been transfected for 24 h were prepared into a cell suspension with serum-free medium. 100 μl of cell suspension containing 1 × 10^4^ cells was added to the upper chamber, and 600 μl of medium containing 10% FBS was added to the lower chamber. After culturing for 24 h, the residual cells on the upper chamber were removed and washed with PBS. Then the cells on the lower surface of the chamber were fixed with paraformaldehyde for 15 min and stained with 0.1% crystal violet for 5 min. After washed with PBS, the cells were photographed and counted under a microscope.

### Western blot

Total proteins were extracted using RIPA buffer, and quantified with a BCA Protein Assay Kit (Tiangen, Beijing, China). 20 μg of proteins were taken for SDS-PAGE electrophoresis, and then electrotransferred onto a PVDF membrane. The membrane was blocked with 5% non-fat milk for 1 h, incubated with the specific primary antibody at 4 °C overnight and incubated with the second antibody for 1 h at room temperature. ECL development was performed after washing the membrane through TBST.

The primary antibodies used in this study were as follow: anti-Bcl2 (1:1000, ab32124), anti-Bax (1:1000, ab32503), anti-p53 (1:1000, ab32389), anti-E-cadherin (1:1000, ab194982), anti-SNAL2 (1:1000, ab180714) and anti-eIF5A (1:1000, ab32443) were rabbit monoclonal antibodies and purchased from Abcam (Cambridge, MA, USA). Anti-Caspase3 (1:1000, ab13847) and anti-N-cadherin (1:1000, ab18203) were rabbit polyclonal antibody and purchased from Abcam. Anti-GAPDH (1:500, ab8245, Abcam) and anti-Vimentin (1:1000, ab8978) were mouse monoclonal antibodies and purchased from Abcam.

### Luciferase reporter assay

The complete 3'UTR of human eIF5A mRNA containing the putative or mutated miR-4640-5p binding site, and the wild or mutated full-length sequence of OGFRP1 were amplified and cloned into the psiCHECK2 vector (Promega). According to the manufacturer's guidelines, Lipofectamine 2000 was used to co-transfect psiCHECK2 recombinant vector and miR-4640-5p mimic or miR-NC into A549 cells. The relative activity of luciferase was measured using the Dual-Luciferase Reporter Assay System (Promega) and the Infinate M200 PRO microplate reader (Tecan, Shanghai, China).

### Statistical analysis

All data were statistically analyzed using SPSS software version 22.0 (IBM Corp., Armonk, NY). All results were expressed as mean ± standard deviation. The difference between groups was calculated using the Student's t-test or one-way ANOVA. P < 0.05 was considered statistically significant.

## Results

### OGFRP1 is high expressed in LUAD tissues and be a prognostic marker

**Fig. 1:**
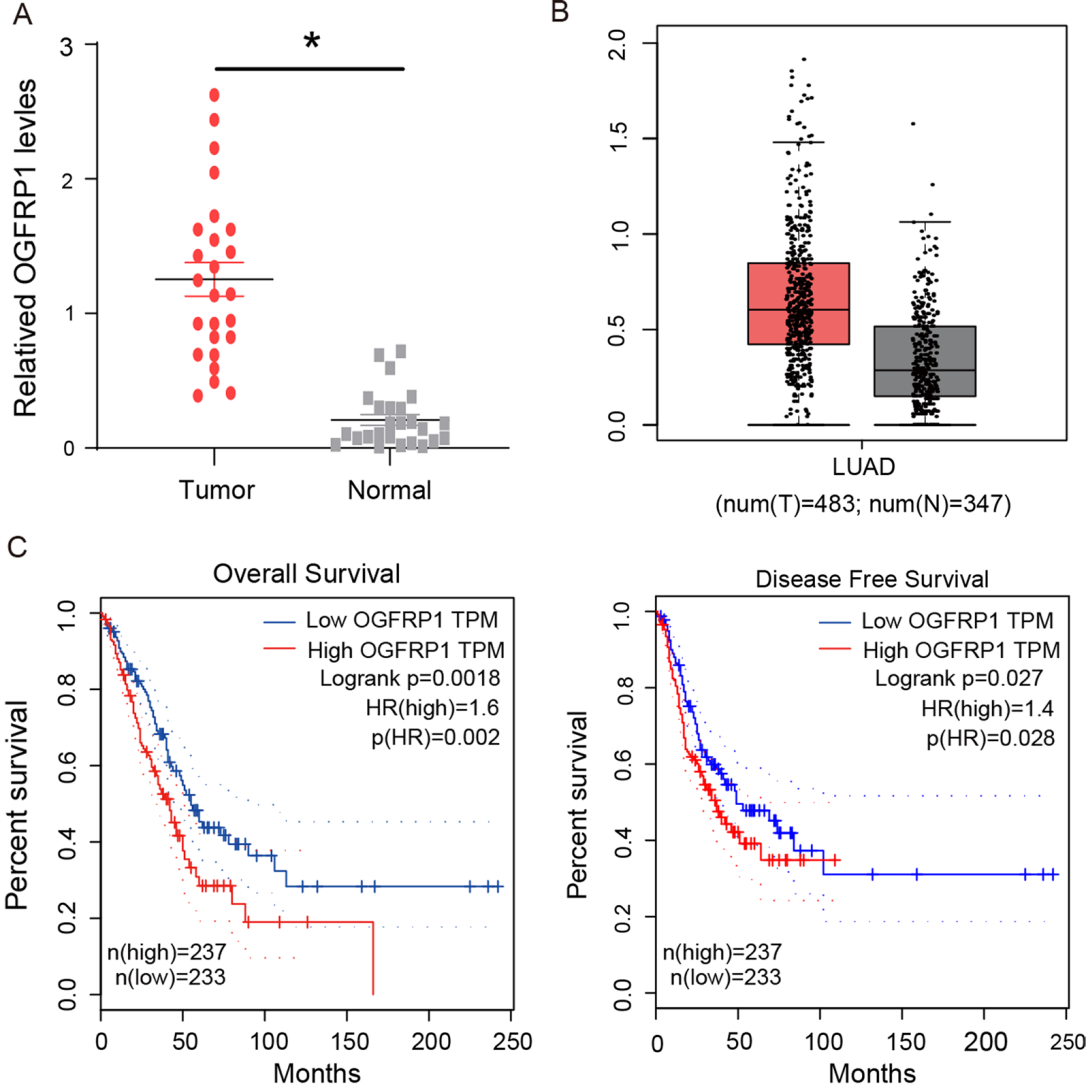
OGFRP1 is high expressed in LUAD tissues and be a prognostic marker. **A** The expression levels of OGFRP1 in 25 pairs of clinically collected LUAD (Tumor) and normal lung tissue samples. **B** The boxplot of OGFRP1 transcriptional expression in LUAD and normal lung tissues. The red and gray boxes represent LUAD and normal tissues respectively. The y-axis indicated the log2-transformed gene expression level. **C** Overall and disease-free survival curves of LUAD patients with different OGFRP1 expression. The result of B and C derived from GEPIA (http://gepia.cancer-pku.cn/), which was based on the database of TCGA and GTEx. * represented P < 0.05

First, the difference in the expression level of OGFRP1 in LUAD and normal tissues was investigated. The data comes from clinically collected tissue samples (25 pairs) and GEPIA. As shown in Fig. 1A, the expression levels of OGFRP1 in LUAD tissues (n = 25) were significantly higher than that in normal lung tissue (n = 25) (*P* < 0.05). The data from GEPIA also showed that the expression of OGFRP1 in LUAD tissues (n = 483) was 2 times higher than that in normal controls (n = 347) (Fig. 1B, P < 0.05). The basic expression pattern implied that OGFRP1 might play a positive role in the NSCLC progression. The correlation between OGFRP1 expression and the survival of LUAD patients analyzed on GEPIA was shown in Fig. 1C. The result illustrated that LUAD patients with high OGFRP1 expression had lower overall and disease-free survival rates. The above results suggested that OGFRP1 was involved in the tumorigenesis of NSCLC and could be a potential therapeutic target or poor-prognosis marker for NSCLC treatment.

### Down-regulation of OGFRP1 inhibits the proliferation, migration and invasion of NSCLC cells

**Fig. 2:**
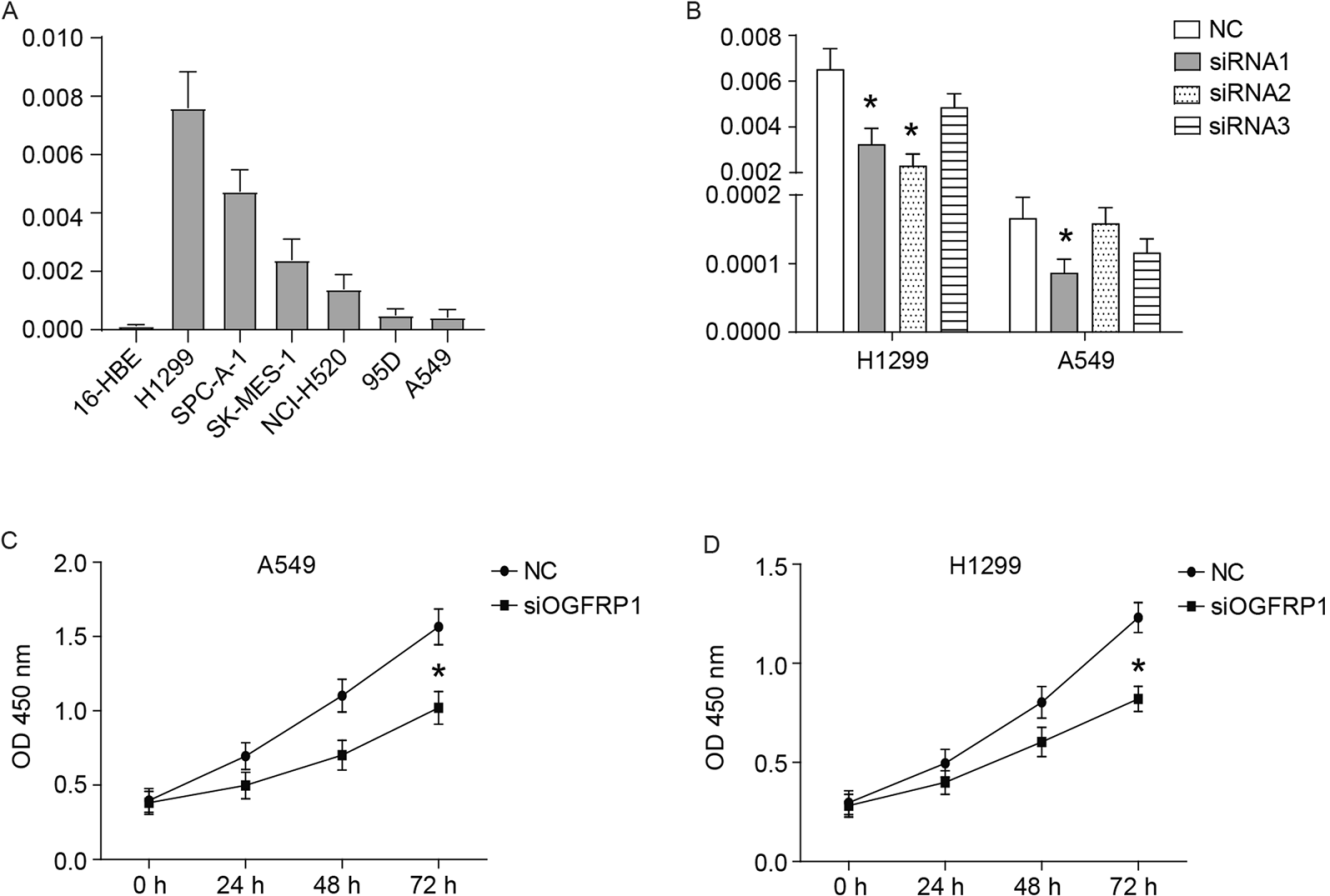
Down-regulation of OGFRP1 inhibits the proliferation of NSCLC cells. **A** The expression level of OGFRP1 in normal human bronchial epithelial cell line 16-HBE and NSCLC cell lines (H1299, SPC-A-1, SK-MES-1, NCI-H520, 95D, A549) was tested by qRT-PCR. **B** Interference efficiency of 3 alternative siRNAs was detected by qRT-PCR. The proliferation of A549 (**C**) and H1299 (**D**) cells transfected with siOGFRP1 was measured by CCK8 assay. * represented P < 0.05

Subsequently, the expression level of OGFRP1 in normal human bronchial epithelial cell line 16-HBE and NSCLC cell lines (H1299, SPC-A-1, SK-MES-1, NCI-H520, 95D, A549) was tested, and the results showed that the expression level of OGFRP1 in all NSCLC cell lines was significantly higher than that in the 16-HBE cell line (Fig. 2A). The H1299 cell line with a higher expression level of OGFRP1 and the A549 cell line with a lower expression level were used for subsequent experimental verification. 3 siRNAs with different target sites were synthesized to down-regulate the expression of OGFRP1 in cells. As shown in Fig. 2B, the most powerful siRNA1 was used in subsequent experiments.

**Fig. 3:**
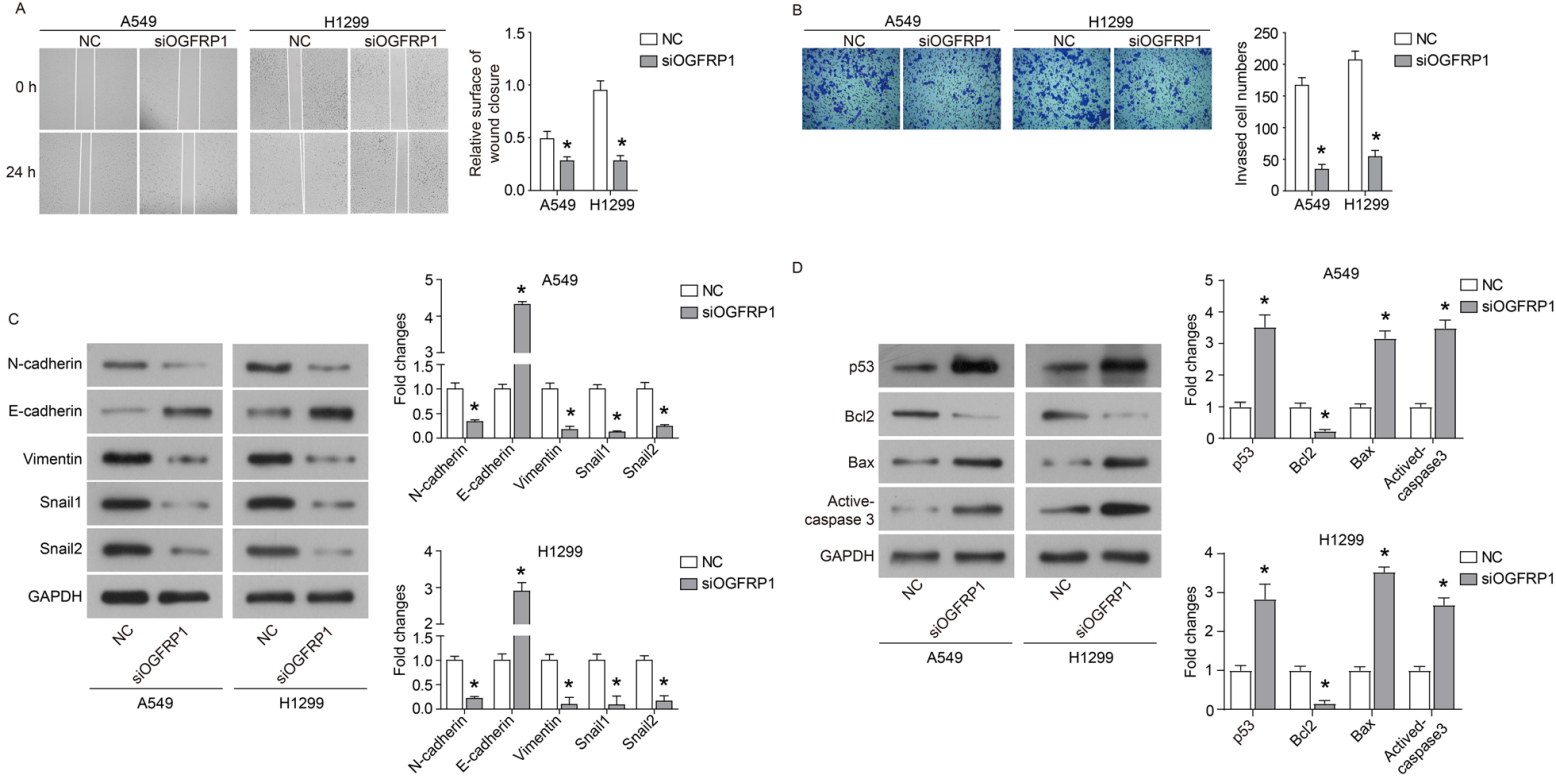
Down-regulation of OGFRP1 inhibits the migration and invasion of NSCLC cells. **A** Cell migration was examined by wound healing assay. The images were taken at 0 h and 24 h after wound formed. **B** Images of invasive cells in transwell assay. The expression of EMT associated proteins (**C**) and apoptosis associated proteins (**D**) was detected by western blot and normalized to GAPDH. * represented P < 0.05

The CCK8 assay was used to detect the effect of siOGFRP1 on the proliferation of NSCLC cells. As shown in Fig. 2C and D, the OD value in 72 h of the siOGFRP1 group was significantly reduced than that in the NC group (*P* < 0.05). Cell migration was investigated by wound healing assay. The result was shown in Fig. 3A, indicating that compared with the NC group, the wound width of the siOGFRP1 group remained relatively greater (*P* < 0.05). The relative migrated area at 24 h (Fig. 3A) also suggested a significant difference between NC and siOGFRP1. Subsequently, cell invasion was investigated by the in vitro Matrigel invasion assay. The result shown in Fig. 3B indicated that the number of invasive NSCLC cells (crystal violet stained) was much lower than that in the NC group (*P* < 0.05). Furthermore, the expression of E-cadherin was up-regulated, while the expression of N-cadherin, Vimentin, Snail1 and Snail2 were down-regulated in siOGFRP1 group (Fig. 3C). In addition, western blotting analysis revealed increased expression of Bax, cleaved caspase 3 and p53, alongside decreased expression of Bcl2 in siOGFRP1 group (Fig. 3D). These results revealed that OGFRP1 played an important role in NSCLC cell proliferation, migration, invasion and apoptosis.

### OGFRP1 directly interacted with miR-4640-5p in NSCLC cells

**Fig. 4:**
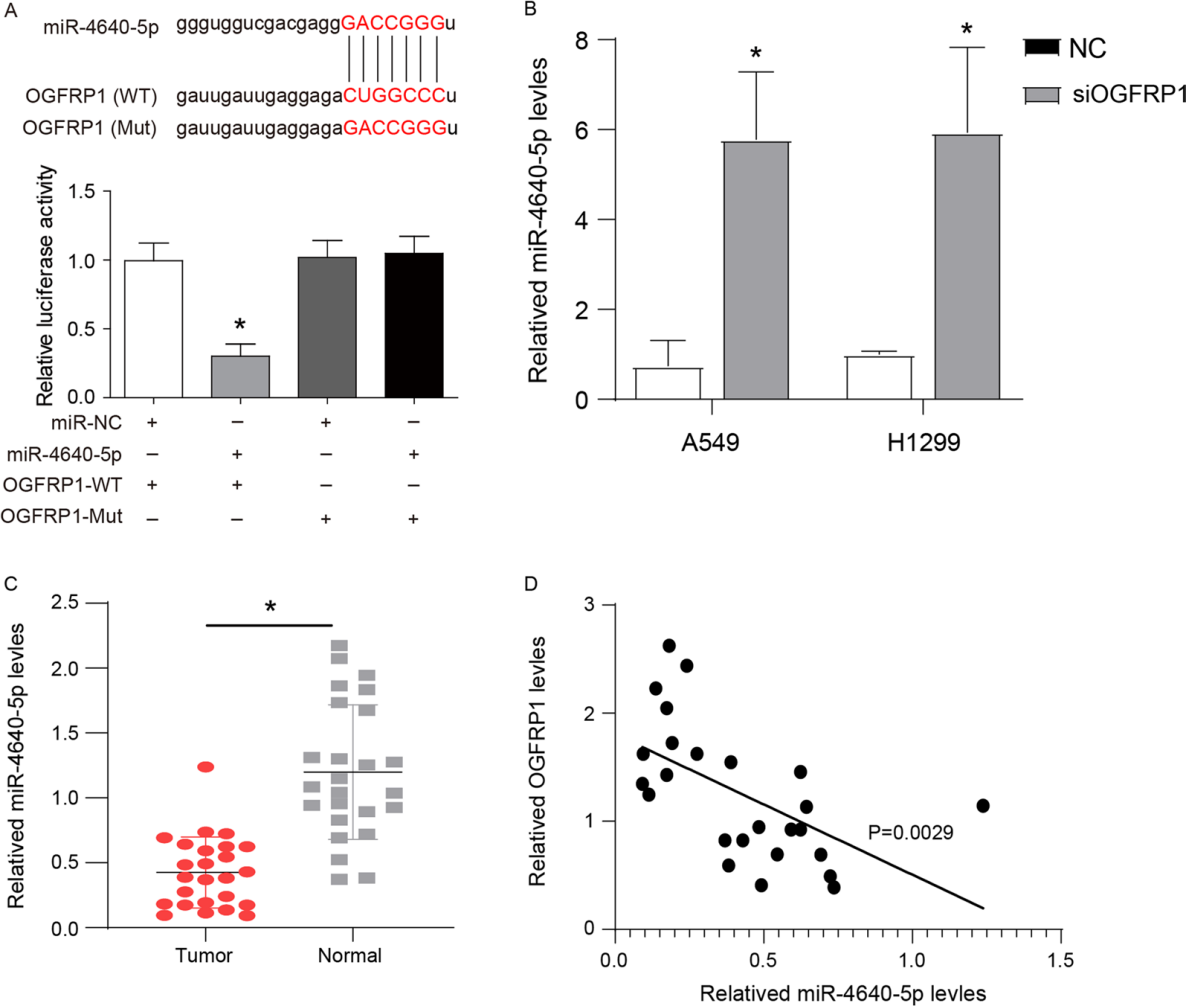
OGFRP1 directly interacted with miR-4640-5p in NSCLC cells. **A** Upper line: The sequences of miR-4640-5p, wide type of OGFRP1 (WT) and mutated OGFRP1 (Mut). Lower line: The expression levels of luciferase of A549 cells transfected with wild-type (WT) or mutated (Mut) OGFRP1 reporters plus miR-4640-5p mimic or miR-NC were determined. **B** The level of miR-4640-5p in A549 and H1299 cells transfected with siOGFRP1 (**C**). The expression levels of miR-4640-5p in 25 pairs of clinically collected LUAD and normal lung tissue samples. **D** The correlation between miR-4640-5p with OGFRP1 levels in 25 pairs of clinically collected LUAD (Tumor) and normal lung tissue samples.* represented P < 0.05

In order to explore the possible molecular mechanism of OGFRP1 function, starBase v2.0 (http://starbase.sysu.edu.cn/) was used to predict a potential miRNA that can directly bind to OGFRP1. Figure 4A showed the binding site of OGFRP1 and miR-4640-5p. The dual luciferase reporter experiment also further proved that OGFRP1 can directly interact with miR-4640-5p. As shown in Fig. 4A, A549 cells co-transfected with miR-4640-5p mimic and OGFRP1-WT showed less luciferase activity than other groups. In addition, down-regulation of OGFRP1 expression significantly increased the level of miR-4640-5p in A549 and H1299 cells (Fig. 4B). A relatively low expression of miR-4640-5p (Fig. 4C) and a significant negative correlation with OGFRP1 levels (Fig. 4D) were found in LUAD tissues. These results demonstrated the direct interaction between OGFRP1 and miR-1243p.

### OGFRP1 exerts its role through regulating miR-4640-5p/eIF5A axis

**Fig. 5:**
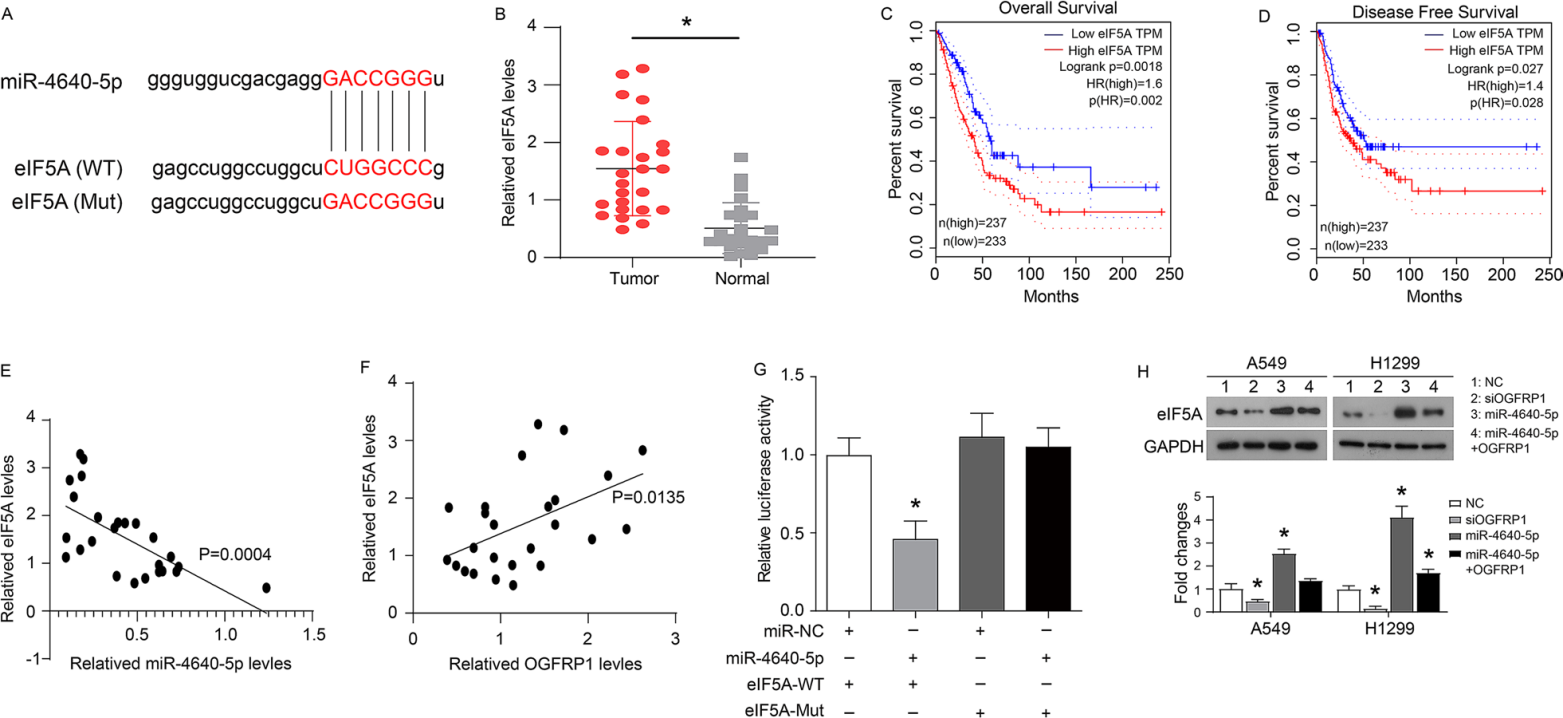
OGFRP1 exerts its role through regulating miR-4640-5p/eIF5A axis. **A** The sequences of miR-4640-5p, wide type of eIF5A (WT) and mutated eIF5A (Mut). **B** The protein expression of eIF5A in 25 pairs of LUAD and normal lung tissues. Overall (**C**) and disease-free (**D**) survival curves of LUAD patients with different eIF5A expression. The result of **C** and **D** derived from GEPIA (http://gepia.cancer-pku.cn/), which was based on the database of TCGA and GTEx. **E** The correlation between miR-4640-5p with eIF5A levels in 25 pairs of clinically collected LUAD and normal lung tissue samples. **F** The correlation between OGFRP1 with eIF5A levels in 25 pairs of clinically collected LUAD and normal lung tissue samples. **G** The expression levels of luciferase of A549 cells transfected with wild-type (WT) or mutated (Mut) eIF5A reporters plus miR-4640-5p mimic or miR-NC were determined. **H** The protein expression of eIF5A. * represented P < 0.05

Finally, starBase v2.0 (http://starbase.sysu.edu.cn/) predicted that eIF5A mRNA was the binding target of miR-4640-5p (Fig. 5A). In fact, western blot was used to detect the expression of eIF5A in 25 pairs of LUAD and normal lung tissues, and it was found that eIF5A was significantly highly expressed in LUAD tissues (Fig. 5B). LUAD patients with low eIF5A expression have better overall (Fig. 5C) and disease-free (Fig. 5D) survival. In addition, the expression levels of miR-4640-5p and eIF5A in LUAD tissues were negatively correlated (Fig. 5E), while the expression levels of OGFRP1 and eIF5A were positively correlated (Fig. 5F).

The dual luciferase report further verified the direct binding of miR-4640-5p and eIF5A mRNA 3'UTR (Fig. 5G). It was observed that A549 cells co-transfected with miR-4640-5p mimic and eIF5A-WT showed less luciferase activity than other groups (Fig. 5G). Moreover, knockdown of OGFRP1 inhibited the expression of eIF5A (Fig. 5H). Transfection of miR-4640-5p inhibitor up-regulated the expression of eIF5A, while knockdown of OGFRP1 reversed the expression of eIF5A (Fig. 5H). These data indicated that eIF5A was the target gene of miR-4640-5p and OGFRP1.

## Discussion

As the development of RNA sequencing technology, thousands of lncRNAs have been identified, which account for the majority of genome transcripts and regulate a large range of cell processes [12, 29–31]. In terms of cancer, lncRNAs have been found to play an important role in cancer progression in vitro and in vivo [16, 32–35]. However, the functionally annotated lncRNAs account for a small part of the total lncRNAs. More research is needed on the function of lncRNAs, especially those with important prognostic and therapeutic values.

In this study, we aimed to determine the functions and its underlying mechanisms of OGFRP1 in NSCLC. We found that expression of OGFRP1 in LUAD was up-regulated and negatively correlated with the survival rate of patients, indicating that OGFRP1 might be a prognostic biomarker or a therapeutic target. Then we screened a most effective siRNA (siOGFRP1) from the 3 candidates to inhibit the expression of OGFRP1, and examined the effect on A549 and H1299 cells. siOGFRP1 could significantly inhibit the proliferation, migration and invasion of A549 and H1299 cells. In addition, siOGFRP1 transfection changed the expression of EMT-related and apoptosis-related proteins. These data revealed the oncogene function of OGFRP1 in NSCLC, which was consistent with the findings in endometrial cancer [22], hepatocellular carcinoma [24], gestational choriocarcinoma cells (JEG3) [25] and cervical carcinoma cells [23].

Eukaryotic translation initiation factor 5A (eIF5A) is an 18-kDa protein that participates in mRNA-related functions, such as transcription [36, 37], mRNA turnover [38] and nucleoplasmic transport [39], plays a role in the initiation and extension of protein synthesis [40, 41], which is essential for cell proliferation. According to reports, eIF5A is highly expressed in a variety of tumors, and is associated with poor clinical features and prognosis, including lung adenocarcinoma [41]. In lung tumor tissues, eIF5A is observed in both the cytoplasm and the nucleus [41]. In the present study, we also found the high expression of eIF5A in LUAD tissues and its correlation with prognosis.

Furthermore, we found that OGFRP1 could act as a ceRNA to target and regulate eIF5A expression through miR-4640-5p. As a ceRNA, lncRNA can bind a variety of different miRNAs. For example, Tang et al. find that OGFRP1 regulates LYPD3 expression by sponging miR-124-3p [28]. According the report, the binding sites of OGFRP1 to miR-4640-5p and miR-124 are not consistent. We believe that there are still other unknown miRNAs that interact with OGFRP1. At present, the regulation mechanism of eIF5A gene expression has not been fully determined. In lung cancer, the increased expression of eIF5A protein is associated with the oncogenic mutations of *K-ras* at codons 12 and 13 [41], which indicates that the *K-Ras* signaling pathway induces eIF5A expression. Treatment of Bcr-Abl^+^ K562 cells with imatinib (a drug that inhibits Abl tyrosine kinase) can reduce eIF5A protein and mRNA levels [42]. This finding indicates that eIF5A may also be induced by the Bcr-Abl oncogene. Considering the incomplete correlation between eIF5A mRNA and protein levels, this may also mean that there is translation control or other post-transcriptional regulatory mechanisms. A mechanism based on the E3 ubiquitin ligase CHIP/Stub1 to induce protein degradation has been reported [43]. In addition to oncogene-driven transcription and post-transcriptional regulation, our study reported the epigenetic regulation of eIF5A by ceRNA for the first time.

## Conclusion

In conclusion, we found that OGFRP1 might be a prognostic biomarker, and the down-regulation of OGFRP1 inhibited progression of NSCLC by regulating eIF5A expression. Our research suggested that OGFRP1 may be a potential molecular target for future NSCLC treatment.

## Data Availability

The data supporting the conclusions of this paper are included within the manuscript.
